# Out-of-Pocket Expenditure and Catastrophic Health Spending Among Patients With Type 2 Diabetes in Rural Tamil Nadu: A Cross-Sectional Study

**DOI:** 10.7759/cureus.106182

**Published:** 2026-03-31

**Authors:** Ganapathy Narayana Arul M, Meriton Stanly A

**Affiliations:** 1 Community Medicine, Sri Ramachandra Institute of Higher Education and Research, Chennai, IND

**Keywords:** catastrophic health expenditure, diabetes mellitus type 2, health policy and economics, noncommunicable diseases (ncds), out-of-pocket expenditure

## Abstract

Background and objectives

Diabetes mellitus is one of the most predominant noncommunicable diseases (NCDs) globally as well as in India. Apart from morbidity and mortality, it causes a significant burden on the healthcare resources of the nation as well as the households. High expenses toward healthcare can potentially impoverish a household. Hence, this study was conducted to assess the economic burden involved in the management of diabetes among the rural population.

Materials and methods

The study was conducted in the Rural Health and Training Centre of Sri Ramachandra Medical College. Data was collected using a semistructured questionnaire, regarding their sociodemographic details, duration of illness, comorbidities, glycemic levels, complications of diabetes, compliance to treatment, and all direct and indirect expenses for the management of diabetes mellitus in the last 3 months.

Results

The mean quarterly out-of-pocket expenditure (OOPE) was 1159 ± 1374₹, in which direct expenses were 727 ± 1124₹, and indirect expenses came to around 413 ± 712₹. Catastrophic health expenditure (CHE) was present in 6.6% (3.65-10.83) of them. The mean expenditure was significantly lower in people who availed government and other free or subsidized healthcare services, and higher in those who were on insulin therapy.

Conclusion

Our existing health system and policies need to be further strengthened, and health coverage and schemes should be made accessible to a larger population to avoid adverse financial implications.

## Introduction

One of the most predominant noncommunicable diseases (NCDs) globally, as well as in our country, is diabetes mellitus. According to the National Family Health Survey-5 (NFHS-5) data, the prevalence of diabetes mellitus was 15.6% and 13.5% for men and women, respectively [1]. Apart from morbidity and mortality, it causes a significant burden on our healthcare services. It affects the nation’s economy and quality of life.

The annual medical expenses among individuals with diabetes are nearly two times those of individuals without diabetes mellitus, and this financial burden is more severe in the cases of developing nations like India [2]. The cost implications of diabetes need to be studied in more detail, as most frequently the costs associated with the management of diabetes are unaffordable to many people, especially those living in developing and underdeveloped countries. This cost increases with the increase in the number of hospitalizations with complications and comorbidities [3]. Healthcare-related costs are most frequently met from out of pocket in our country, with the prevalence of out-of-pocket expenditure (OOPE) in healthcare being 62.4% [4].

Catastrophic health expenditures (CHEs) have been defined as health-related expenses that exceed a share of either total income, expenses other than food, or costs toward their essential dietary requirements [4]. Healthcare-related expenditures can potentially impoverish a household. Goal 3 of the United Nations’ Sustainable Development Goals agenda intends to provide Universal Health Coverage (UHC) to its population, alongside improving financial protection. UHC involves enabling access to high-quality healthcare and safe, cost-effective medicines and immunizations for everyone [4]. Aside from the direct and indirect expenses, diabetes mellitus causes a lot of anxiety, fear, suffering, and pain due to its complications, which are often intangible. With proper policy planning and implementation, the government plans to reduce the burden of OOPE among the population and improve healthcare equity.

The primary objective of this study was to estimate the mean OOPE incurred in the management of type 2 diabetes mellitus among patients attending a rural health and training center. The secondary objectives were to assess the factors associated with OOPE among patients with type 2 diabetes mellitus and to estimate the prevalence of CHE among the study population. 

## Materials and methods

Study design

A cross-sectional study was performed in the Rural Health and Training Centre of Sri Ramachandra Medical College, in Vayalanallur, Chennai. It was conducted over a period of four months (September 2023-December 2023).

Patient recruitment and sample size

The study was conducted in patients with type 2 diabetes mellitus and aged above 18 years attending the outpatient department (OPD) of the center and willing to provide informed consent. Patients who were newly diagnosed (within three months before the interview), sick patients, and those with other forms of diabetes were excluded from the study. The calculated sample size based on estimation of the mean, with 95% CI using the mean and standard deviation from previous studies, was 212 [5]. A consecutive sampling technique was used, where all the eligible patients attending the OPD were included in the study till the sample size was met. 

Data collection

A signed informed consent was obtained from the patients or their caregivers, who were willing to participate in the study. Information was collected from patients or their attenders, using a semistructured questionnaire and face-to-face interview, followed by general and systemic examination. Data were collected regarding their sociodemographic details, duration of illness, glycemic levels, complications of diabetes, compliance with treatment, comorbidities, and all direct and indirect expenses for the management of diabetes mellitus in the last three months. Total income was assessed based on the self-reported total monthly income of all earning members in the household. All the expenses, including doctor consultations, investigations, hospitalizations, transportation, and miscellaneous expenses for both the patient and their caregiver, were recorded. Physical bill copies were verified wherever they were available.

Statistical analysis

Data was entered using EpiCollect 5 and MS Excel (Microsoft Corporation, Redmond, Washington, United States) and analyzed using SPSS Statistics for Windows, Version 16 (Released 2007; SPSS Inc., Chicago, Illinois, United States). Descriptive statistics were summarized using mean, standard deviation, median, and percentage. For analytical statistics, univariate and multivariate linear regression were used. Variables used for linear regression were age, gender, occupation, type of health facility preferred, and medication used. Multivariate linear regression was performed to adjust for potential confounding variables.

Ethical approval

This study was conducted after getting approval from the Institutional Ethics Committee of Sri Ramachandra Medical College. (IEC reference number-CSP-MED/24/JUL/106/214)

Definition of Terms

Out-of-pocket expenditure: Amount spent for the management of diabetes mellitus alone, including the direct and indirect expenditures, for both the patient and the caregivers, in the last three months.

Direct expenditure: This includes the expenses toward doctor consultation charges, laboratory investigations, medicines, and disposables, in the last three months.

Indirect expenditure: This includes the expenses like transport, food, and loss of wages, for both the patient and the caregivers, in the last three months.

Catastrophic health expenditure: OOPE is termed as catastrophic when the expenditure is more than 10% of the family income in the last three months [6].

## Results

**Figure 1:**
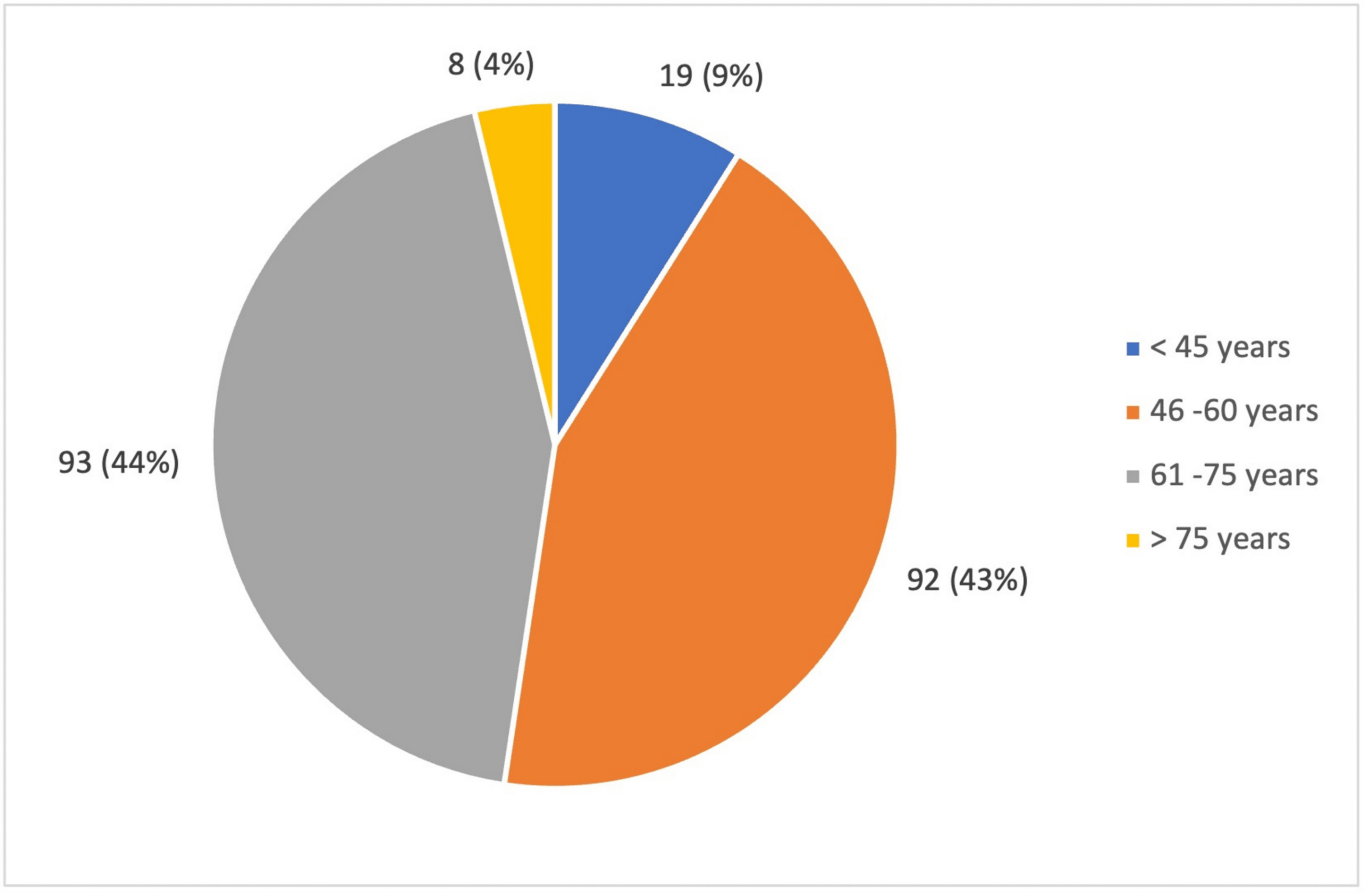
Age distribution among the study population (in years) This figure shows the distribution of study participants (n = 212) across different age groups

**Figure 2:**
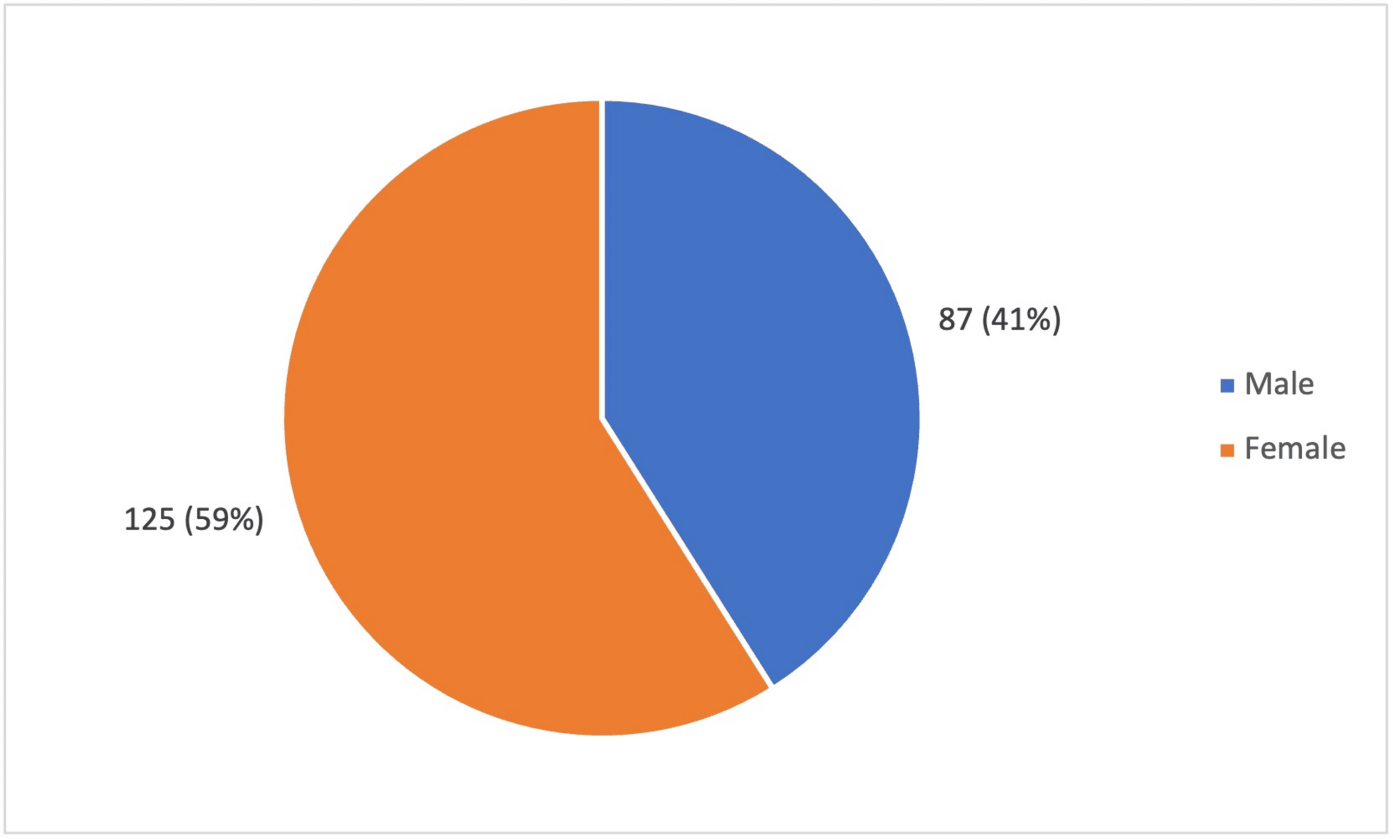
Sex distribution among the study population This figure illustrates the gender distribution of the study population (n = 212)

**Figure 3:**
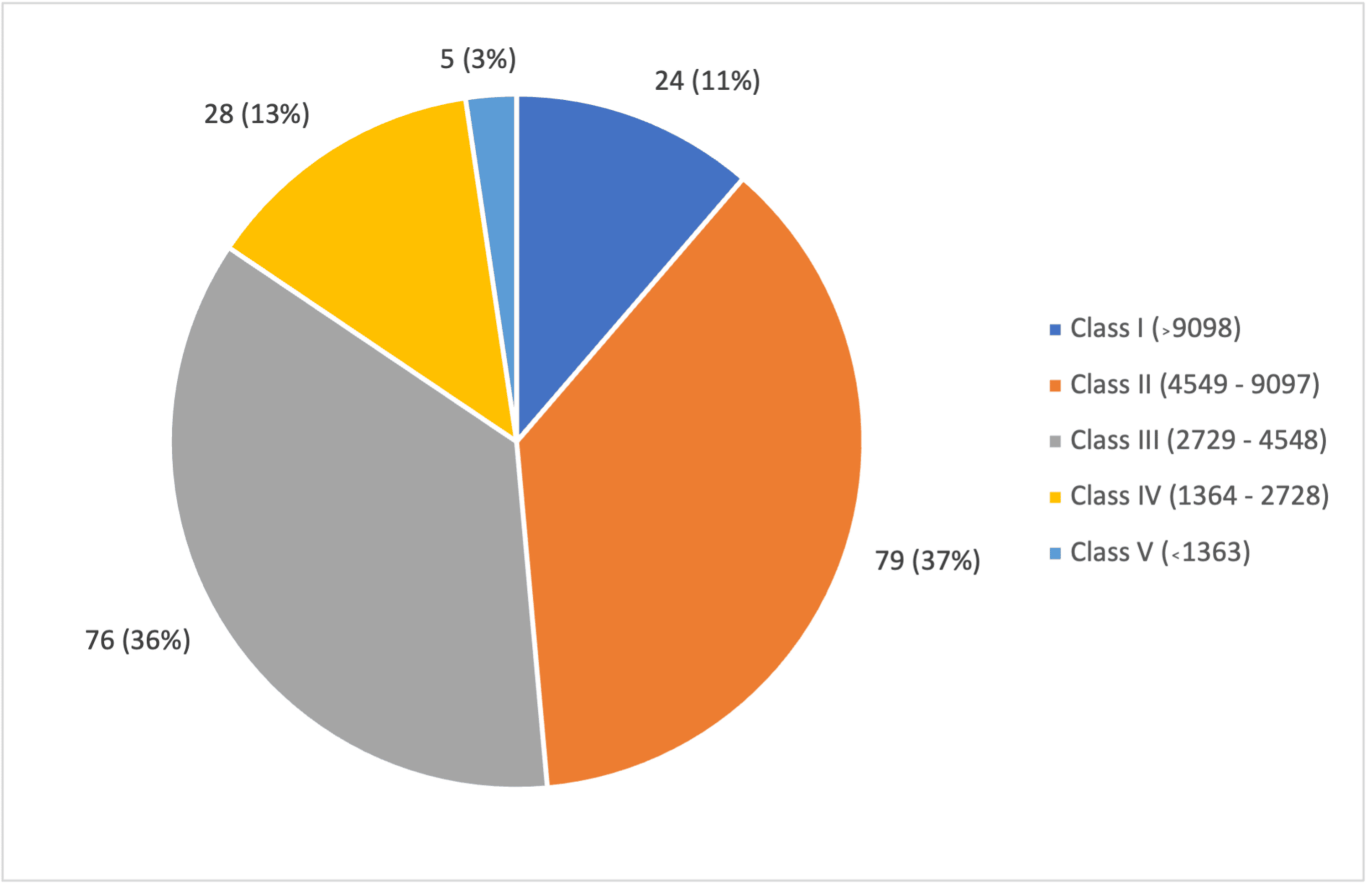
Socioeconomic status distribution among the study population This figure shows the distribution of participants (n = 212) according to socioeconomic status based on the Modified BG Prasad Scale (2024)

In the study, the mean age of the patients was 58 ± 10 years (Figure 1). Out of the 212 participants, 125 (59%) were female, and 87 (41 %) were male (Figure 2). The majority of the participants belonged to Class II and Class III of the socioeconomic scale, according to the Modified BG Prasad Scale 2024 (Figure 3). Among the participants, 66 (31.1%) studied only up to primary school, and 44 did not have any formal education. The mean fasting blood sugar (FBS) was 143 ± 61 mg/dL, and the post-prandial blood sugar value was 244 ± 186 mg/dL. A total of 84 (39.6%) participants had other comorbid conditions like dyslipidemia, hypertension, thyroid disorders, coronary artery disease, etc. Most of the study participants (96.2%) were managed using oral hypoglycemic agents, and only a few of them (3.8%) were on insulin therapy.

The mean and median quarterly OOPE are 1159 ± 1374₹ and 410₹, respectively (Table 1). Out of the total expenditure, direct costs, which included doctor consultations, medicines, laboratory investigations, etc., were around 727 ± 1124₹, and indirect costs, which included transportation, loss of wages for both patients and caregivers, were around 413 ± 712₹. 

**Table 1:** Mean OOPE OOPE: out-of-pocket expenditure; SD: standard deviation This table shows the mean quarterly OOPE incurred by patients with type 2 diabetes mellitus (n = 212) in Indian Rupee (INR)

	Mean expenditure (in INR) ± SD (median)
Total costs	1159 ± 1374 (410)
Total direct costs	727 ± 1124 (100)
Total indirect costs	413 ± 712 (180)

The total expenditure in patients who sought government and other subsidized health facilities was 552 ± 935₹, less than that of those who used private facilities, whose total expenditure was around 1486 ± 1466₹ (p <0.001). Among participants who utilize the subsidies provided by the government of Tamil Nadu, the mean total quarterly OOPE was approximately 807 ± 1112₹ (Table 2). Patients who used insulin also showed significantly higher OOPE than those who were managed with oral hypoglycemic agents. Age, gender, and occupation did not show any statistically significant association. 

**Table 2:** Mean quarterly out-of-pocket expenditure among participants (n = 212) with and without government subsidies SD: standard deviation This table compares the mean quarterly out-of-pocket expenditure in Indian Rupee (INR) among participants with and without government subsidy utilization

	Mean quarterly out-of-pocket expenditure ± SD (median) without government subsidies (n = 170)	Mean quarterly out-of-pocket expenditure ± SD (median) with government subsidies (n = 42)
Total costs	1242 ± 1423 (510)	807 ± 1112 (170)
Direct costs	744 ± 1152 (120)	626 ± 1009 (0)
Indirect costs	493 ± 782 (225)	98 ± 201 (0)

**Figure 4:**
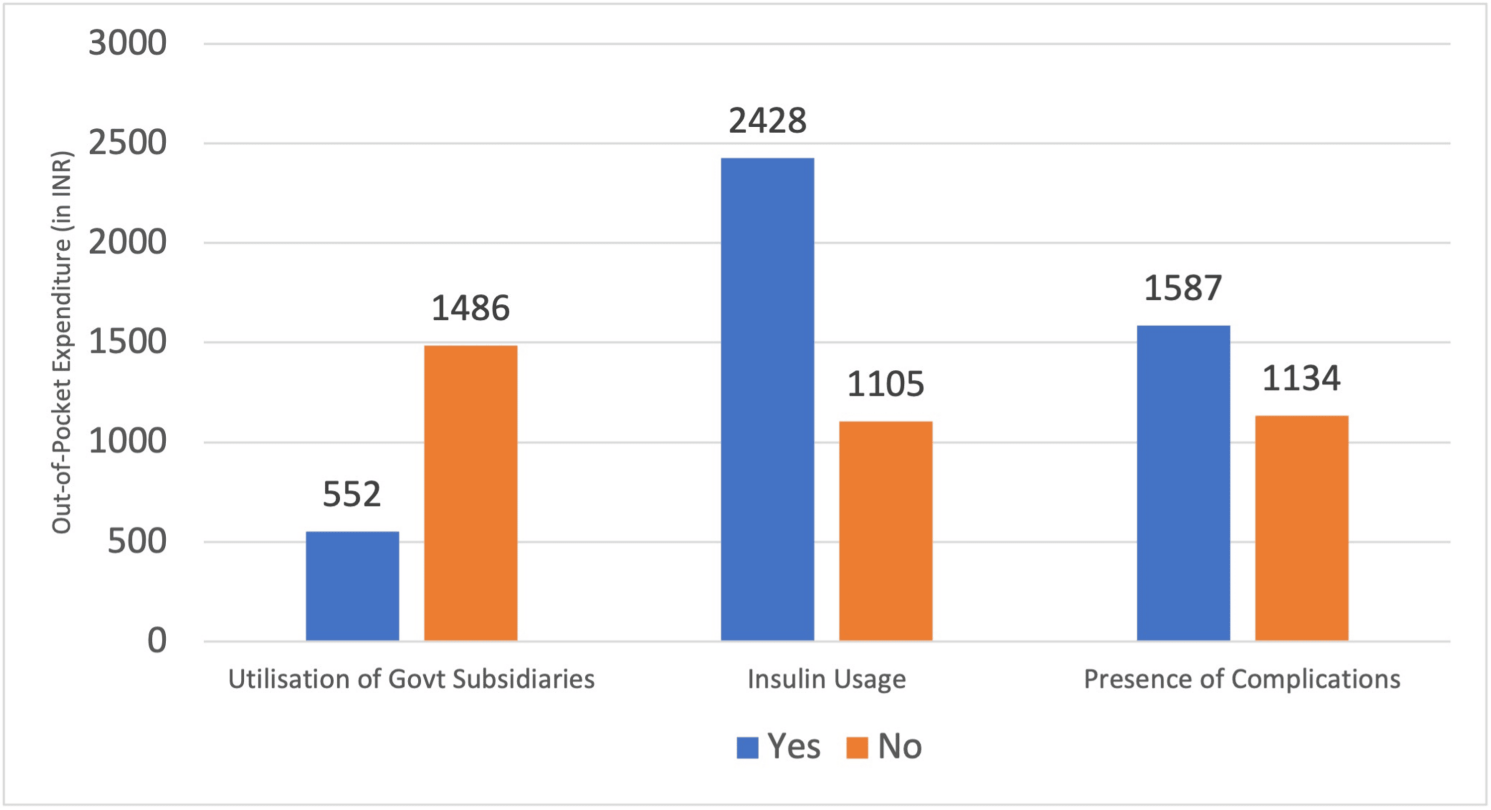
Out-of-pocket expenditure across different determinants This figure depicts variation in out-of-pocket expenditure among different subgroups, in Indian Rupee (INR)

**Figure 5:**
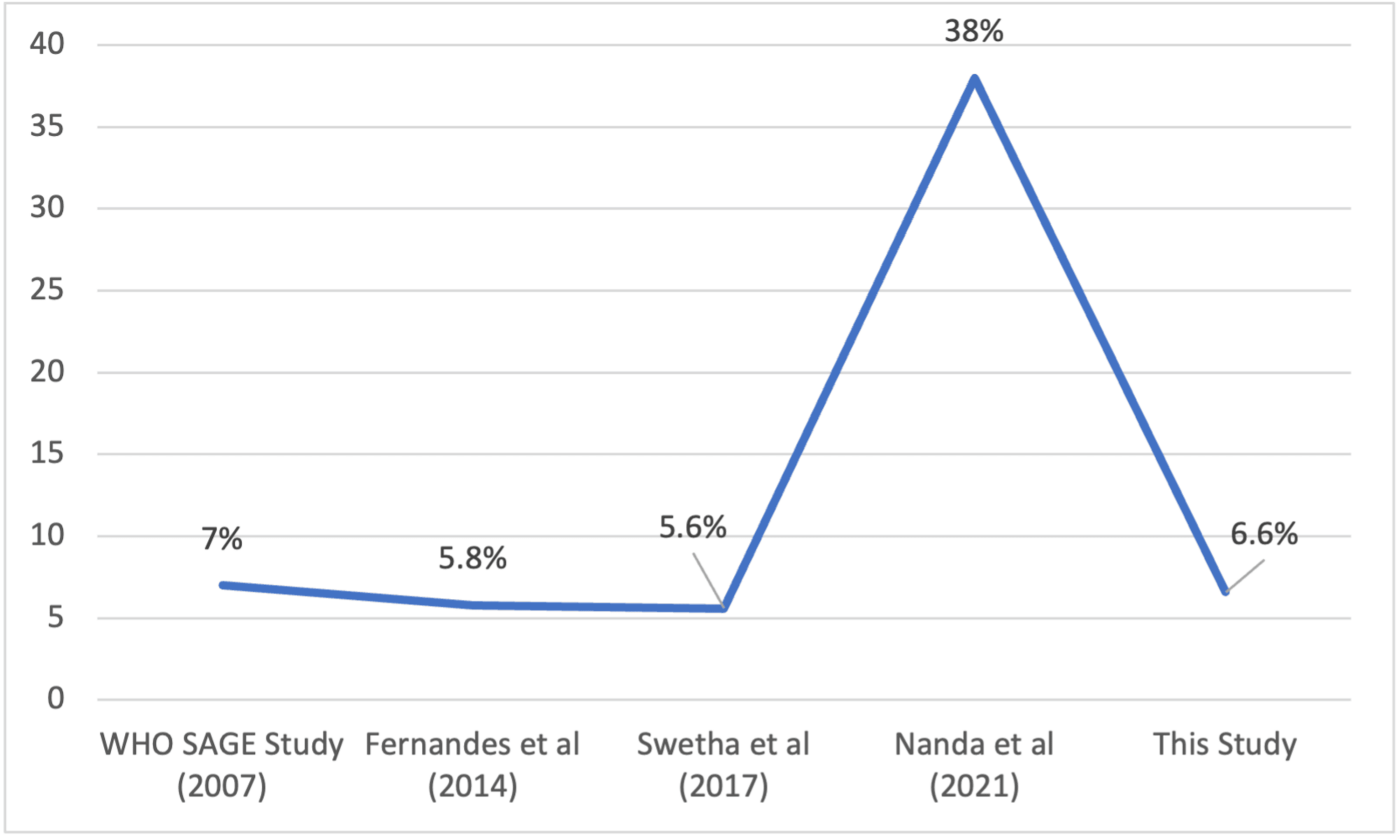
Trend in CHE (in %) CHE: catastrophic health expenditure This figure illustrates the trend in the prevalence of CHE reported in studies conducted over time

The mean quarterly OOPE among various variables is depicted in Figure 4. On linear regression analysis, the type of medication used and the health facility preferred were found to be associated with higher OOPE (Table 3). Most of the study participants spent less than 10% of their income toward the medical expenses, except 6.6% (3.65-10.83) of them, who had CHE due to the disease (Figure 5). 

**Table 3:** Association between out-of-pocket expenditure and study variables using linear regression analysis This table shows the association between out-of-pocket expenditure and study variables using univariate and multivariate linear regression analysis

	β	95% CI		Adjusted β	95% CI		p
		Lower	Upper		Lower	Upper	
Age	-27.9	-45.8	-10.1	-16.2	-35.8	3.5	0.06
Gender	58.0	-321.5	437.5	-176.7	-575.9	222.5	0.46
Occupation	159.2	-80.7	399.2	419.2	-24.8	863.2	0.07
Type of medication	1322.8	359.6	2285.9	935.8	17.7	1853.9	0.04
Health facility preferred	933.6	564.4	1302.9	867.0	496.2	1237.9	0.001

## Discussion

The present study was conducted to estimate the OOPE incurred in the management of type 2 diabetes mellitus and to assess the factors associated with it, along with the prevalence of CHE among patients attending a rural health and training center.

More than 60% of the patients belonged to Class II and Class III of the socioeconomic scale, as it was conducted in a predominantly rural population. This is also supplemented by the fact that the majority of the participants have only studied up to primary school or less.

The mean total quarterly OOPE was found to be 1159 ± 1374₹, which is consistent with findings in other studies by Eshwari et al. (5041 annually) [2], Pushparani et al. (quarterly OOPE (IQR): 1719₹ (2958₹) [7], Pati et al. (2653₹ ± 2795₹) [8], and Saima Nazir et al. (408.12₹ ± 23.37₹ [9]. Mean costs were higher in this study than in the one conducted by Basu et al. [3], which can be attributed to the fact that the study was carried out exclusively in a government tertiary care center.

The expenditure was found to be lower in those who utilized the schemes provided by the government of Tamil Nadu, like the Zero Bus Ticket Travel for women passengers, Chief Minister Comprehensive Health Insurance Scheme (CMCHIS), Makkalai Thedi Maruthuvam (MTM), and Tamil Nadu Old Age Pension Scheme (TNOAPS), and central government schemes like Ayushman Bharat-Pradhan Mantri Jan Arogya Yojana (AB-PMJAY) and Indira Gandhi Old Age Pension Scheme (IGNOAPS). Median direct and indirect costs were negligible in them, since most of the costs were covered by the subsidies provided by the government (Table 2). 

The factors associated with higher OOPE were the health facility available and medications given, which correlated with the results of studies by Ambade et al. [10]. Other variables like age, gender, and occupation did not show any statistically significant association with OOPE.

The mean expenditure was significantly lower in people who availed government and other free healthcare services than those who sought private healthcare (p-value < 0.001). Patient’s choice of preferring private healthcare facilities despite the government providing free healthcare could be influenced by factors such as accessibility, perceived quality of care, availability of medications, etc. [11].

The quarterly OOPE varied significantly with the type of medicine being prescribed for diabetes. It was very high in the people who take insulin for treatment (p-value < 0.001). This might be explained by the fact that most of the patients who use insulin rely on the private sector to get the medicines, which was also found in a study done by Priya et al. in Puducherry [12]. Aside from the insulin itself, many other costs come along with insulin therapy, such as disposables like insulin syringes, and for self -glucose monitoring. Apart from the costs involved, regular insulin injections also cause significant mental agony and pain and impair the quality of life of both the patient and their caregivers.

OOPE was found to be lower in females than in males, and those without any complications due to diabetes, but these differences were not statistically significant. Hence, the factors that were associated with higher OOPE in diabetes mellitus were found to be the type of health facility used and the treatment modality.

This study took 10% of income as the threshold, beyond which was deemed as CHE. Nanda et al. in their study had multiple threshold points for CHE, at 10%, 20%, and 30%. The prevalences of CHE at each of the threshold points were 37.9%, 20.3%, and 8.8%, respectively, in that study [13]. The prevalence of CHE was around 6.6%, which was found to be lower than in other studies [14]. This can be partly explained by various health schemes provided by the government. CHE has been on an alarming rising trend in the past decade, with rising healthcare costs, concomitant loss of jobs and income due to the effect of the COVID-19 pandemic, where the CHE rose to 37% in 2021, from being around 7% in 2007 [13-16].

This study does have some limitations. The study was conducted at a single rural health and training center; the findings may not be generalizable to the entire rural population. Only the patients who attended the OPD were included, and those who required hospitalization at the time of study were not included. The exclusion of severely ill or hospitalized patients might have resulted in an underestimation of OOPE. Even though the answers of the patients were verified as far as possible, there exists some recall bias, as patients would have answered for the past three months based on memory. Additionally, the use of consecutive sampling may introduce selection bias. Since the study is cross-sectional in nature, a causal relationship between OOPE and the determinants could not be established. 

## Conclusions

To conclude, many studies, including this one, highlight the financial burden due to the management of diabetes. An extensive burden falls upon the economically vulnerable population, who might become impoverished over the course of the treatment of diabetes and its complications. Even though both the state government and central government have been doing a commendable job, our existing health system could be further strengthened, and health coverage and schemes could be made accessible to a larger population. Targeted outreach programs and health education initiatives may help improve awareness and utilization of government healthcare services and welfare schemes among vulnerable populations and reduce the financial burden. 
